# Determining the status of small dense low-density lipoprotein cholesterol level in women undergoing menopausal transition

**DOI:** 10.3389/fendo.2024.1500712

**Published:** 2025-01-20

**Authors:** Qingfang He, Yujia Fang, Lixin Wang, Mingbin Liang, Xiangyu Chen, Ruying Hu, Jieming Zhong

**Affiliations:** Department of Chronic Non-Communicable Disease Control and Prevention, Zhejiang Provincial Center for Disease Control and Prevention, Hangzhou, China

**Keywords:** small dense low-density lipoprotein cholesterol, premenopause, perimenopause, postmenopause, menopausal transition

## Abstract

**Objective:**

Research on small dense low-density lipoprotein cholesterol (sdLDL-C) and menopausal status remains scarce. Our aim is to evaluate the relationship between serum sdLDL-C level and different menopausal status in a Chinese women population.

**Methods:**

In 2022, a cross-sectional study was conducted including electronic standardized questionnaire surveys, anthropometric measurements, and biological specimen examinations based on communities. Permanent resident adults aged 30–69 years who lived in two communities in Zhejiang Province and participated in a community health examination from May 26 to September 17 were recruited. According to their menopausal status, the eligible women subjects were divided into premenopause, perimenopause, and postmenopause. Logistic regression model by SAS software was used to explore the association with sdLDL-C level and different menopausal status.

**Results:**

A total of 2,062 women subjects were included with a median age of 57 (51, 63) years. There were 451 (21.9%) premenopause, 87 (4.2%) perimenopause, and 1,524 (73.9%) postmenopause women. The median sdLDL-C level was 0.937 (0.685, 1.209) mmol/L, and the sdLDL-C levels showed a gradually and significantly upward trend in premenopause, perimenopause, and postmenopause women, and this peaked in the postmenopause women. Logistic regression analysis showed that after controlling the confounding factors, compared with premenopause, postmenopause was significantly associated with increased sdLDL-C concentration (OR = 1.514, 95% CI: 1.025–2.238), while no significant association was observed either between serum sdLDL-C and perimenopause (compared with premenopause) or between serum sdLDL-C and postmenopause (compared with perimenopause).

**Conclusions:**

An elevated sdLDL-C level was significantly associated with postmenopause and independent of chronological aging. The study supports that sdLDL-C is a promising risk biomarker for menopausal transition. Future studies should consider a broader population and a more rigorous and thorough study design to validate these findings.

## 1 Introduction

The life expectancy at birth in mainland China was 81.0 years for women in 2019 and is expected to rise steadily and reach 85.1 years for women by 2035 (1). However, the age of ovarian function decline in women has not changed, and the average menopause age is still around 48 years old (2), which means that at least one-third or more of a woman’s life is spent in menopause and postmenopause. Perimenopause is a period that women must undergo from fertile to old, and it is also the beginning period of various chronic diseases, especially cardiovascular and cerebrovascular diseases (3, 4). Early reports (3, 5) had shown that the incidence of atherosclerosis, cerebral infarction, and coronary heart disease in premenopause women was lower than that in men of the same age, and the onset of cardiovascular disease (CVD) in women was about 10 years later than that in men. However, the prevalence of CVD in postmenopausal women significantly increased, and the advantage which they had against men disappeared after the age of 60. This phenomenon might be related to a decrease in estrogen levels in women (5).

Estrogen deficiency after menopause might cause dyslipidemia, abnormal glucose metabolism, elevated blood pressure, sympathetic tension, impaired endothelial function, and vascular inflammation (6). Hyperlipidemia is considered one of the most important risk factors for CVD (3). At the beginning of perimenopause, women’s blood lipid profile begins to change, mainly manifested by the increase of total cholesterol (TC), low density lipoprotein cholesterol (LDL-C), and triglyceride (TG) levels. Dyslipidemia in women increased significantly after menopause. In China, TC and LDL-C levels in women over 50 years old were significantly higher than those in men of the same age (7). A study of Women’s Health Across the Nation (SWAN) found that TC, LDL-C, and apolipoprotein B (Apo B) demonstrated substantial increases within the 1-year interval before and after menopause, independent of the age of menopause, which could further lead to endothelial injury and atherosclerosis (8). The serum LDL-C level remains the primary target of lipid-lowering therapy (9, 10); however, many individuals with normal LDL-C levels still develop CVD (11, 12), and the residual risk may be partially conferred by lipoprotein entities such as the small dense low density lipoprotein (sdLDL) particles (13).

Plasma LDL particles had polydispersity, LDL particles with density greater than 1.034 g/mL and diameter less than 25.5 nm are collectively referred to as sdLDL, and the cholesterol they carry is named small dense low-density lipoprotein cholesterol (sdLDL-C) (14). The genesis of sdLDL is closely linked to the states of hypertriglyceridemia. Berneis and Kaspar have proposed two different pathways for sdLDL formation based on hepatic triglyceride availability (15). Hypertriglyceridemia generates TG-rich very-low-density lipoprotein (VLDL). VLDL is firstly hydrolyzed by lipoprotein lipase and then mediated by cholesteryl ester transfer protein (CETP). The TG of VLDL is exchanged with the cholesteryl ester of LDL and high-density lipoprotein (HDL) particles to produce TG-rich LDL, which is finally delipidated by hepatic lipase and converted into sdLDL-C. Recent studies demonstrated that elevated levels of sdLDL-C predicted the risk of developing coronary heart disease (CHD), even in individuals whose LDL-C levels were considered to be at a low CVD risk (10, 16). At present, there are few studies on the changes of blood lipid profile, especially sdLDL-C, before and after the menopause. This study was to compare the sdLDL-C and other blood lipid levels among premenopause, perimenopause, and postmenopause women so as to identify the abnormals early and timely and to adopt appropriate intervention measures to reduce the risk of CVD.

## 2 Materials and methods

### 2.1 Participants

In 2022, a cross-sectional study was conducted including electronic standardized questionnaire surveys, anthropometric measurements, and biological specimen examinations based on communities. Permanent resident adults who lived in two communities in Zhejiang Province and participated in a community health checkup from May 26 to September 17 were recruited as participants. The inclusion criteria were as follows: 30–69 years, conscious, no mental illness, and able to complete the questionnaire independently. Subjects who meet any of the following items during the survey period were excluded: dementia, schizophrenia, ill in bed, and pregnant or lactating women.

In total, 4,222 subjects with complete survey data were obtained. We excluded subjects step by step as follows: ever diagnosed with dyslipidemia and taking lipid-lowering drugs and/or ever diagnosed with diabetes and taking hypoglycemic drugs (*n* = 652), male (*n* = 1,473), and use of hormone replacement therapy (*n* = 34), and 2,062 female subjects who met the inclusion and exclusion criteria were finally included.

This study was approved by the Ethics Committee of Zhejiang Provincial Center for Disease Control and Prevention (2020-040-01). Each participant signed a written informed consent.

### 2.2 Methods

An electronic survey system was developed for the electronic standardized questionnaire surveys and the storage of data on questionnaires, anthropometric measurements, and biological specimen examinations.

#### 2.2.1 Measurement of blood samples

Three tubes of fasting venous blood samples were obtained from each subject: one sodium fluoride anticoagulant tube, one gel serum tube, and one ethylenediaminetetraacetic acid (EDTA) anticoagulant tube. All of the blood samples should be stored at 2°C–8°C before they were measured. Plasma sample from the sodium fluoride anticoagulant tube was provided for fasting blood glucose (FBG; maccure: CH0404102: hexokinase method). The serum sample from the gel serum tube should be left for at least half an hour, centrifuged within 2 h, and then be provided for sdLDL-C (BSBE GSSD/B: peroxidase method), total cholesterol (TC; maccura CH0404152: oxidase method), triglyceride (TG; maccura CH0404151: enzymatic method), low-density lipoprotein cholesterol (LDL-C; maccura CH0404162: direct clearance method), and high-density lipoprotein cholesterol (HDL-C; maccura CH0404161: direct clearance method). All of the measurements above were measured by using an automatic biochemical analyzer.

Blood specimen from the EDTA anticoagulant tube was provided for glycated hemoglobin A1c (HbA1c; ARKRAY 101656: high-performance liquid chromatography) by using an automatic HbA1c analyzer and hemoglobin (mindray 105-001961-00: colorimetric method) by using an automatic hematology analyzer.

All of the measurements above should be finished by KingMed Diagnostics (Hangzhou) Co., Ltd. on the same day as the blood samples were collected, and all of the results should be uploaded to the electronic survey system on the same day.

#### 2.2.2 Data collection and medical examination

Electronic standardized questionnaires were conducted through face-to-face interviews by using tablet computers. Social demographic characteristics (such as age, gender, and education level), behaviors and lifestyle (such as smoking, drinking, and physical activity), history of major chronic diseases (such as diabetes, hyperlipidemia, hypertension, and medication use), history of female fertility (such as age of menarche, number of pregnancies, cesarean, oral contraceptive use, IUD (intrauterine device) use, hysterectomy, ovariosteresis, and lumpectomy of breast tumor), and life quality data (such as sleep quality, nap habits, life satisfaction, etc.) were collected.

Physical examination was performed after overnight fasting for at least 8 h, including height, fasting weight, fasting waist circumference (WC), systolic blood pressure (SBP), and diastolic blood pressure (DBP). Height and weight were measured using a scale, accurate to 0.1 cm and 0.1 kg, respectively. To measure the waist circumference, a waist circumference gauge accurate to 0.1 cm was used. Blood pressure was measured with an electronic sphygmomanometer (Omron, Shanghai, China) accurate to 1 mmHg in two consecutive measurements (interval 1 min) on the right arm and averaged.

All of the physical examination results above should be uploaded to the electronic survey system on the same day.

#### 2.2.3 Definition criteria

According to the standards of medical care for type 2 diabetes in China in 2019 (17), diabetes mellitus is defined as FBG ≥7.0 mmol/L and/or approved by a healthcare provider’s diagnosis.

Hypertension refers to systolic blood pressure (SBP) ≥140 mmHg and/or diastolic blood pressure (DBP) ≥90 mmHg and/or who had previously been diagnosed with hypertension according to the 2018 Chinese guidelines for the management of hypertension (18).

According to the 2016 Chinese guideline for the management of dyslipidemia in adults (19), any of the following is defined as dyslipidemia: TC ≥5.2 mmol/L and/or TG ≥1.70 mmol/L and/or LDL-C ≥3.4 mmol/L and/or HDL-C <1.0 mmol/L and/or those who had previously been diagnosed with dyslipidemia.

Body mass index (BMI) = weight (kg)/(height (m) ^2^) according to WS/T 428-2013 Criteria of Weight for Adults (20).

Smoking is defined as smoking more than one cigarette per day for 6 months (consecutive or cumulative) (21).

Drinking refers to a response of ≥1 time a week in the past year; alcoholic beverages included beer, liquor, red wine, and rice wine (22).

Regular physical activity is defined as ≥150 min per week of moderate-intensity exercise or a combination of moderate- and high-intensity exercise or ≥75 min per week of high-intensity exercise (23).

Premenopause was defined as regular menstruation. Women whose menstrual bleeding had spontaneously ceased for more than 38 days but less than 12 months were classified as perimenopause. Having 12 consecutive months of amenorrhea was defined as postmenopause, including surgical or natural menopause (24).

Number of pregnancies is the sum of the number of live births, stillbirths, spontaneous abortions, and induced abortions, including ectopic pregnancies.

Oral contraceptive use is defined as continuous use for at least 3 months.

IUD use is defined as continuous placement of IUD for at least 3 months.

Sleep quality: The participants were divided into two groups—good sleep quality and poor sleep quality—according to their answer to the question “in general, how do you think of your sleep quality for the recent 1 month, good or poor?”

### 2.3 Quality control

The investigators all went through unified centralized training and qualification before the investigation. All interviews and examinations were conducted according to standardized protocols. The Provincial Center for Disease Control and Prevention and the on-site investigation team jointly set up a quality control team to strictly control on-site questionnaires, physical measurements, laboratory tests, and information entry.

### 2.4 Statistical analysis

Statistical analyses were performed using SAS software (version 9.4, SAS Institute Inc., Cary, NC, USA). Continuous variables were tested for normality, and normal distribution data were described as *x* ± s, and the comparison between groups was performed by *t*-test and analysis of variance. Data with abnormal distribution were represented by M(Q1, Q3), and a comparison between groups was performed using non-parametric tests (such as rank-sum test). Furthermore, *χ* ^2^ test and Wilcoxon rank-sum test were used for group comparisons of categorical and rank variables, respectively. Multivariate logistic regression was used to analyze the relationship between menopausal status and sdLDL-C level, age, education years, drinking, regular physical activity, BMI and WC, hypertension, SBP, DBP, age of menarche, number of pregnancies, cesarean, cumulative years of oral contraceptive use, etc. *P*-values <0.05 were considered statistically significant.

## 3 Results

### 3.1 General characteristics

The 2,062 female subjects’ median age was 57 (51, 63) years. The number (%) of premenopause, perimenopause, and postmenopause women was 451 (21.9%), 87 (4.2%), and 1,524 (73.9%), respectively. The prevalence of dyslipidemia in premenopause, perimenopause, and postmenopause women was 54.5%, 74.7%, and 79.9%, respectively. There were only seven smoking subjects, so smoking was not included in the analysis. Compared with premenopause subjects, perimenopause and postmenopause women were much older (*P* < 0.001), with much later age of menarche (*P* < 0.001), with much less pregnancies (*P* = 0.025) and percentage of cesarean (*P* < 0.001), with much higher percentage of less than 9 years of education (*P* < 0.001), never used oral contraceptives (*P* < 0.001), had hysterectomy (*P* < 0.001), had poor sleep quality (*P* = 0.010), and unsatisfied with life (*P* = 0.002), with much higher prevalence of dyslipidemia (*P* < 0.001) and hypertension (*P* < 0.001), and with much higher (*P* < 0.001) DBP, SBP, WC, TC, TG, LDL-C, sdLDL-C, sdLDL-C/LDL-C, sdLDL-C/HDL-C, FBG, HbA1c, and hemoglobin levels. Postmenopause women had the highest HDL-C concentration (1.46 mmol/L vs. 1.41 mmol/L (premenopause) and 1.34 mmol/L (perimenopause), *P* = 0.008). Perimenopause women had the highest rate of drinking (14.9% vs. 8.6% (premenopause) and 5.2% (postmenopause), *P* < 0.001). The sdLDL-C levels showed a gradually and significantly upward trend in premenopause (0.806 mmol/L), perimenopause (0.943 mmol/L), and postmenopause women (0.972 mmol/L) and peaked in postmenopause women (*P* < 0.001). The specific characteristics of the research subjects are shown in **Table 1**.

**Table 1:** Demographic characteristics of the 2,062 participants at different menopausal status.

Characteristics	All(n=2062)	Premenopause(n=451)	Perimenopause(n=87)	Postmenopause(n=1524)	*χ^2^/F/H*	*P* value
Age [years, *M*(*Q*_1_,*Q*_3_)]	57 (51, 63)	45 (39, 49)	51 (49, 55)	60 (56, 64)	914.989	0.000**
Education years (<9 years), *n(%*)_1_	1892 (91.8)	377 (83.6)	80 (92.0)	1435 (94.2)	51.385	0.000**
Regullar physical activity, *n(%)*_1_	94 (4.6)	23 (5.1)	3 (3.4)	68 (4.5)	0.583	0.747
Drinking, *n(%)*_1_	132 (6.4)	39 (8.6)	13 (14.9)	80 (5.2)	17.765	0.000**
Number of pregnancies [*n*, *M*(*Q*_1_,*Q*_3_)]	3(2,3)	3(2,4)	3(2,3)	3(2,3)	7.376	0.025*
Age of menarche [years, *M*(*Q*_1_,*Q*_3_)]	16(15,17)	15 (14,16)	15 (14,17)	16 (15,18)	187.973	0.000**
Cesarean, *n(%)*_1_	249 (12.1)	162 (35.9)	18 (20.7)	69 (4.5)	329.368	0.000**
Never took oral contraceptive, *n(%)*_1_	1875 (90.9)	358 (79.4)	76 (87.4)	1441 (94.6)	98.585	0.000**
Cumulative years of oral contraceptive use, *n(%)*_1_					98.834	0.000**
No	1875 (90.9)	358 (79.4)	76 (87.4)	1441 (94.6)		
< 5	139 (6.7)	68 (15.1)	8 (9.2)	63 (4.1)		
≥ 5	48 (2.3)	25 (5.5)	3 (3.4)	20 (1.3)		
Never placed IUD, *n(%)*_1_	835 (40.5)	159 (35.3)	30 (34.5)	646 (42.4)	8.712	0.013
Hysterectomy, *n(%)*_1_	146 (7.1)	4 (0.9)	4 (4.6)	138 (9.1)	36.143	0.000**
Ovariosteresis_2_, *n(%)*_1_	27 (1.3)	6 (1.3)	2 (2.3)	19 (1.2)	0.707	0.702
Lumpectomy of the breast tumor, *n(%)*_1_	56 (2.7)	10 (2.2)	2 (2.3)	44 (2.9)	0.651	0.722
Good sleep quality, *n(%)*_1_	1048 (50.8)	283 (62.7)	48 (55.2)	717 (47.0)	35.019	0.010**
Nap habits, *n(%)*_1_	1516 (73.5)	327 (72.5)	57 (65.5)	1132 (74.3)	3.551	0.169
Satisfied with life, *n(%)*_1_	1254 (60.8)	302 (67.0)	43 (49.4)	909 (59.6)	12.762	0.002**
Hypertension, *n(%)*_1_	1103 (53.5)	138 (30.6)	48 (55.2)	917 (60.2)	122.434	0.000**
DBP [mmHg, *M*(*Q*_1_,*Q*_3_)]	82 (76, 90)	80(72, 86)	84(76, 92)	84(76, 90)	39.081	0.000**
SBP [mmHg, *M*(*Q*_1_,*Q*_3_)]	134 (120, 148)	122 (112, 135)	132 (120, 146)	137 (124, 150)	190.110	0.000**
BMI [kg/m^2^, *M*(*Q*_1_,*Q*_3_)]	23.4 (21.4, 25.7)	23.4 (21.2, 25.9)	23.4 (21.4, 26.0)	23.4 (21.5, 25.6)	0.161	0.923
WC [cm, *M*(*Q*_1_,*Q*_3_)]	83.1 (77.2, 89.0)	80.1 (75.1, 87.1)	81.4 (76.0, 87.1)	84.0 (78.3, 89.1)	39.350	0.000**
TC [mmol/L, *M*(*Q*_1_,*Q*_3_)]	5.42 (4.78, 6.09)	4.90(4.38, 5.59)	5.35 (4.67, 5.84)	5.57 (4.98, 6.26)	140.578	0.000**
TG [mmol/L, *M*(*Q*_1_,*Q*_3_)]	1.41 (1.02, 2.02)	1.19(0.83, 1.79)	1.59 (0.97, 2.23)	1.46 (1.08, 2.07)	48.206	0.000**
HDL-C [mmol/L, *M*(*Q*_1_,*Q*_3_)]	1.45 (1.24, 1.70)	1.41(1.19, 1.68)	1.34 (1.22, 1.60)	1.46 (1.25, 1.72)	9.624	0.008**
LDL-C [mmol/L, *M*(*Q*_1_,*Q*_3_)]	3.30 (2.73, 3.87)	2.92(2.49, 3.55)	3.18 (2.58, 3.70)	3.38 (2.83, 3.95)	72.888	0.000**
Dyslipidemia_3_, *n(%)*_1_	1529 (74.2)	246 (54.5)	65 (74.7)	1218 (79.9)	116.932	0.000**
sdLDL−C_4_ [mmol/L, *M*(*Q*_1_,*Q*_3_)]	0.937 (0.685, 1.209)	0.806 (0.580, 1.086)	0.943 (0.634, 1.320)	0.972 (0.713, 1.228)	56.208	0.000**
sdLDL−C/LDL−C*_5_* [*M*(*Q*_1_,*Q*_3_)]	0.28 (0.23, 0.34)	0.27 (0.22, 0.33)	0.30 (0.23, 0.34)	0.29 (0.24, 0.34)	17.904	0.000**
sdLDL−C/HDL−C_6_ [*M*(*Q*_1_,*Q*_3_)]	0.66 (0.44, 0.93)	0.56(0.36, 0.87)	0.73 (0.44, 0.98)	0.67 (0.46, 0.94)	22.588	0.000**
FBG_7_ [mmol/L, *M*(*Q*_1_,*Q*_3_)]	5.39(5.07, 5.82)	5.17(4.86, 5.50)	5.26 (4.93, 5.74)	5.45 (5.15, 5.89)	114.163	0.000**
HbA1c_8_ [%, *M*(*Q*_1_,*Q*_3_)]	5.8(5.5, 6.0)	5.6 (5.3, 5.8)	5.8 (5.5, 6.0)	5.8 (5.6, 6.1)	154.663	0.000**
Hemoglobin_9_ [g/L, *M*(*Q*_1_,*Q*_3_)]	136(129, 143)	134 (124, 140)	135 (131, 143)	137 (130, 143)	41.666	0.000**

*: significance level <0.05; **: significance level <0.01.

1. n (%): frequency (n) and percentage (%). 2. Ovariosteresis: removal of ovaries (unilateral or bilateral). 3. Dyslipidemia: according to reference (19), any of the following is defined as dyslipidemia: TC ≥5.2 mmol/L; and/or TG ≥1.70 mmol/L; and/or LDL-C ≥3.4 mmol/L; and/or HDL-C <1.0 mmol/L; and/or those who had previously been diagnosed with dyslipidemia. 4. sdLDL−C: small dense low-density lipoprotein cholesterol. 5. sdLDL−C/LDL−C: the ratio of sdLDL-C to LDL−C. 6. sdLDL−C/HDL−C: the ratio of sdLDL-C to HDL−C. 7. FBG: fasting blood glucose. 8. HbA1c: glycated hemoglobin A1c. 9. hemoglobin: haemoglobin.

### 3.2 Basic information on the quartile levels of sdLDL-C

The median sdLDL-C concentration of the 2,062 respondents was 0.937 (0.685, 1.209) mmol/L. When all of the 2,062 subjects were divided into four groups (Q1, Q2, Q3, and Q4) according to the quartiles of sdLDL-C, we found that compared with Q1, the median ages (*P* < 0.001), age of menarche (*P* = 0.007), proportion of postmenopause (*P* < 0.001), and prevalence of dyslipidemia (*P* < 0.001) and hypertension (*P* < 0.001) of the Q2, Q3, and Q4 groups were significantly gradually increased, while the proportion of cesarean cases significantly gradually decreased (*P* = 0.001). The Q4 group had a significantly higher proportion of drinking (9.6% vs. 4.8% (Q1), 6.2% (Q2), and 5.0% (Q3), *P* = 0.006). The Q2 and Q4 groups had a significantly high proportion of hysterectomy (9.3% (Q2) and 8.6% (Q4) vs. 5.2% (Q1) and 5.2% (Q3), *P* = 0.011). Meanwhile, the levels of DBP, SBP, BMI, WC, TC, TG, LDL-C, sdLDL-C/LDL-C, sdLDL-C/HDL-C, FBG, HbA1c, and hemoglobin all increased with the increase of the sdLDL-C concentration (*P* < 0.001), while HDL-C decreased with the increase of sdLDL-C concentration except Q4 (*P*<0.001) (**Table 2**).

**Table 2:** Basic information of quartiles of sdLDL-C levels of the 2,062 participants.

Characteristics	Q1 (n=516)	Q2 (n=516)	Q3 (n=518)	Q4 (n=512)	*χ^2^/F/H*	*P* value
Age [years, *M*(*Q*_1_,*Q*_3_)]	55 (46, 62)	57 (51, 462)	58 (53, 64)	58 (54, 64)	54.262	0.000**
Education years (<9 years), *n(%)*_1_	462 (89.5)	470 (91.1)	480 (92.7)	480 (93.8)	6.928	0.074
Regullar physical activity, *n(%)*_1_	27 (5.2)	20 (3.9)	25 (4.8)	22 (43)	1.257	0.739
Drinking, *n(%)*_1_	25 (4.8)	32 (6.2)	26 (5.0)	49 (9.6)	12.353	0.006**
Number of pregnancies [*n*,*M*(*Q*_1_,*Q*_3_)]	3 (2,3)	3(2,3)	3(2,3)	3(2,3)	4.033	0.258
Age of menarche [years, *M*(*Q*_1_,*Q*_3_)]	16 (15,17)	16 (15,17)	16 (15,17)	16 (15,17)	12.011	0.007**
Menopausal Status, *n(%)*_1_					59.911	0.000**
Premenopause	167 (32.4)	123 (23.8)	84 (16.2)	77 (15.0)		
Perimenopause	22 (4.3)	20 (3.9)	19 (3.7)	26 (5.1)		
Postmenopause	327 (63.4)	373 (72.3)	415 (80.1)	409 (79.9)		
Cesarean, *n(%)*_1_	87 (16.9)	61 (11.8)	57 (11.0)	44 (8.6)	17.565	0.001**
Never took oral contraceptive, ***n(%)*_1_**	464 (89.9)	468 (90.7)	483 (93.2)	460 (89.8)	4.763	0.190
Cumulative years of oral contraceptive use, *n(%)*_1_					5.829	0.443
No	464 (89.9)	468 (90.7)	483 (93.2)	460 (89.8)		
< 5	39 (7.6)	34 (6.6)	25 (4.8)	41 (8.0)		
≥ 5	13 (2.5)	14 (2.7)	10 (1.9)	11 (2.1)		
Never placed IUD, *n(%)*_1_	214 (41.5)	200 (38.8)	210 (40.5)	211 (41.2)	0.959	0.811
Hysterectomy, *n(%)*_1_	27 (5.2)	48 (9.3)	27 (5.2)	44 (8.6)	11.080	0.011*
Ovariosteresis_2_, *n(%)*_1_	8 (1.6)	8 (1.6)	5 (1.0)	6 (1.2)	1.013	0.798
Lumpectomy of the breast tumor, *n(%)*_1_	10 (1.9)	18 (3.5)	18 (3.5)	10 (2.0)	4.604	0.203
Good sleep quality, *n(%)*_1_	272 (52.7)	268 (51.9)	253 (48.8)	255 (49.8)	2.020	0.568
Nap habits, *n(%)*_1_	375 (72.7)	385 (74.6)	369 (71.2)	387 (75.6)	3.017	0.389
Satisfied with life, *n(%)*_1_	217 (61.4)	305 (59.1)	335 (64.7)	297 (58.0)	5.640	0.131
Hypertension, *n(%)*_1_	219 (42.4)	275 (53.3)	282 (54.4)	327 (63.9)	47.675	0.000**
SBP [mmHg, *M*(*Q*_1_,*Q*_3_)]	128 (114, 141)	133 (120, 147)	135 (122, 148)	138 (124, 152)	67.151	0.000**
DBP [mmHg, *M*(*Q*_1_,*Q*_3_)]	79 (72, 88)	82 (75, 90)	84 (76, 92)	84 (78, 91)	51.763	0.000**
BMI [kg/m^2^, *M*(*Q*_1_,*Q*_3_)]	22.4 (20.3, 24.7)	23.5 (21.7, 25.9)	23.6 (21.6, 25.7)	24.0 (22.2, 26.2)	75.237	0.000**
WC [cm, *M*(*Q*_1_,*Q*_3_)]	80.1 (74.1, 86.6)	83.3 (77.1, 89.1)	83.1 (78.1, 89.1)	85.1 (80.8, 90.0)	95.349	0.000**
TC [mmol/L, *M*(*Q*_1_,*Q*_3_)]	4.64 (4.08, 5.11)	5.16 (4.71, 5.65)	5.58 (5.17, 6.02)	6.42 (5.95, 6.94)	993.965	0.000**
TG [mmol/L, *M*(*Q*_1_,*Q*_3_)]	0.93 (0.72, 1.20)	1.27 (1.01, 1.72)	1.64 (1.26, 2.19)	1.98 (1.51, 2.60)	647.710	0.000**
HDL-C [mmol/L, *M*(*Q*_1_,*Q*_3_)]	1.62 (1.38, 1.89)	1.46 (1.23, 1.72)	1.35 (1.14, 1.59)	1.39 (1.20, 1.61)	149.216	0.000**
LDL-C [mmol/L, *M*(*Q*_1_,*Q*_3_)]	2.55 (2.18, 2.90)	3.10 (2.73, 3.48)	3.51 (3.05, 3.87)	4.18 (3.72, 4.60)	1052.035	0.000**
Dyslipidemia_3_, *n(%)*_1_	164 (31.8)	369 (71.5)	487 (94.0)	509 (99.4)	762.248	0.000**
sdLDL−C/LDL−C_4_ [*M*(*Q*_1_,*Q*_3_)]	0.21 (0.18, 0.23)	0.26 (0.23, 0.30)	0.30 (0.28, 0.35)	0.36 (0.32, 0.39)	1151.062	0.000**
sdLDL−C/HDL−C_5_ [*M*(*Q*_1_,*Q*_3_)]	0.33 (0.26, 0.40)	0.55 (0.46, 0.67)	0.78 (0.65, 0.93)	1.06 (0.90, 1.25)	1477.654	0.000**
FBG_6_ [mmol/L, *M*(*Q*_1_,*Q*_3_)]	5.25 (4.95, 5.61)	5.33 (5.05, 5.73)	5.44 (5.14, 5.85)	5.56 (5.19, 6.14)	95.518	0.000**
HbA1c_7_ [%, *M*(*Q*_1_,*Q*_3_)]	5.6 (5.4, 5.9)	5.8 (5.5, 6.0)	5.8 (5.6, 6.1)	5.9 (5.7, 6.2)	127.426	0.000**
Hemoglobin_8_ [g/L, *M*(*Q*_1_,*Q*_3_)]	133 (126, 139)	135 (129, 142)	136 (130, 143)	139 (131, 145)	80.090	0.000**

Q1: sdLDLC <=0.685 mmol/L; Q2: 0.685 mmol/L<sdLDLC <= 0.937 mmol/L; Q3: 0.937 mmol/L<sdLDLC <= 1.209 mmol/L; Q4: sdLDLC > 1.209 mmol/L. *: significance level <0.05; **: significance level <0.01. 1. n (%): frequency (n) and percentage (%). 2. Ovariosteresis: removal of ovaries (unilateral or bilateral). 3. Dyslipidemia: according to reference (19), any of the following is defined as dyslipidemia: TC ≥5.2 mmol/L; and/or TG ≥1.70 mmol/L; and/or LDL-C ≥3.4 mmol/L; and/or HDL-C <1.0 mmol/L; and/or those who had previously been diagnosed with dyslipidemia. 4. sdLDL−C/LDL−C: the ratio of sdLDL-C to LDL−C. 5. sdLDL−C/HDL−C: the ratio of sdLDL-C to HDL−C. 6. FBG: fasting blood glucose. 7. HbA1c: glycated hemoglobin A1c. 8. hemoglobin: haemoglobin.

### 3.3 Correlation analysis of sdLDL-C with menopausal status

Logistic regression analyses were performed, taking the premenopause group as the control group, the perimenopause group (no = 0; yes = 1) or the postmenopause group (no = 0; yes = 1) respectively as the dependent variable, and sdLDL-C as the independent variable; or taking the perimenopause group as the control group, the postmenopause group (no = 0; yes = 1) as the dependent variable, and sdLDL-C as the independent variable. After adjusting for confounding variables (age, education level, regular physical activity, drinking, number of pregnancies, age of menarche, cesarean, oral contraceptive use, cumulative years of oral contraceptive use, IUD use, hysterectomy, ovariosteresis, lumpectomy of the breast tumor, sleep quality, nap habits, life satisfaction, hypertension, continuous variable DBP, SBP, BMI, and WC), the results showed that compared with premenopause, postmenopause was significantly related to the increase in sdLDL-C concentration of the subjects (OR = 1.514, 95% CI: 1.025–2.238) (**Table 3**). There was no statistically significant association either between serum sdLDL-C and perimenopause (compared with premenopause) or between serum sdLDL-C and postmenopause (compared with perimenopause).

**Table 3:** Logistic regression analysis of sdLDL-C concentration and different menopausal status.

Menopausal status	Postmenopause (compared with premenopause)			Perimenopause (compared with premenopause)			Postmenopause (compared with perimenopause)		
	*β*	*P*-value	OR (95% CI)	*β*	*P*-value	OR (95% CI)	*β*	*P*-value	OR (95% CI)
Model 0	1.065	0.000**	2.902 (2.140–3.935)	0.699	0.017*	2.011 (1.134–3.564)	0.254	0.392	1.290 (0.720–2.310)
Model 1	0.522	0.003**	1.685 (1.200–2.366)	0.330	0.291	1.391 (0.754–2.564)	0.154	0.609	1.166 (0.647–2.102)
Model 2	0.488	0.006**	1.628 (1.148–2.310)	0.415	0.200	1.514 (0.802–2.857)	0.108	0.728	1.114 (0.608–2.042)
Model 3	0.420	0.021*	1.523 (1.064–2.179)	0.340	0.313	1.405 (0.726–2.717)	0.136	0.663	1.146 (0.621–2.113)
Model 4	0.408	0.038*	1.504 (1.024–2.211)	0.374	0.279	1.454 (0.739–2.862)	0.152	0.638	1.165 (0.617–2.196)
Model 5	0.415	0.037*	1.514 (1.025–2.238)	0.382	0.284	1.466 (0.729–2.948)	0.159	0.625	1.173 (0.620–2.218)

Model 0, crude OR (95% CI); Model 1, Model 0 + adjusted for age and education years; Model 2, Model 1 + adjusted for drinking, regular physical activity, BMI, and WC; Model 3, Model 2 + adjusted for hypertension, SBP, and DBP; Model 4, Model 3 + adjusted for number of pregnancies, age of menarche, cesarean, oral contraceptive use, cumulative years of oral contraceptive use, IUD use, hysterectomy, ovariosteresis, and lumpectomy of the breast tumor; Model 5, Model 4 + adjusted for sleep quality, nap habits, and life satisfaction.

*<0.05 (significance level).

**<0.01 (significance level).

## 4 Discussion

Perimenopause women undergo a decline of ovarian follicle function and a decrease of ovarian hormone production. Studies have found that the pathophysiological changes after menopause are not only limited to endocrine changes but also induce metabolic abnormalities such as dyslipidemia, fat redistribution, elevated blood pressure, and insulin resistance, which may cause changes in vascular structure and lead to an increased risk of CVD (3). Reports on the changes of blood lipid profile, especially sdLDL-C and different menopausal status, remain scarce. This cross-sectional investigation elucidated associations between sdLDL-C and menopausal status in a Chinese women population. The menopausal status included premenopause, perimenopause, and postmenopause. Our analysis revealed a significant association between sdLDL-C levels and postmenopause.

The sdLDL-C and sdLDL-C/LDL-C ratio was distributed differently by age and gender. In our study, the sdLDL-C levels showed a step-by-step and significantly upward trend in the premenopause, perimenopause, and postmenopause groups and peaked in the postmenopause group. The proportions of sdLDL-C quartile Q1, Q2, Q3, and Q4 were also significantly increased in the postmenopause group and peaked in Q3 and Q4. The sdLDL-C/LDL-C ratio was similar between postmenopause and perimenopause women but significantly higher than in premenopause women. Vekic’s study (25) demonstrated that gender and menopausal status had a significant impact on sdLDL-C concentrations, for men and postmenopause women had comparable sdLDL-C concentrations, which were both significantly higher than those of premenopause women. Based on a large representative sample (5,208 participants including 2,397 men and 2,811 women) from a Japanese general population, Izumida T et al. described that the sdLDL-C and sdLDL-C/LDL-C ratio was differently distributed by age, gender, and menopausal status. In postmenopause women, the sdLDL-C level and sdLDL-C/LDL-C ratio were significantly higher than in premenopause women, until approximately 65 years, followed by a downward or pleated trend (26). At menopause the LDL particle size distribution shifts toward sdLDL particles and increases the sdLDL-C levels (27), which is due to increased hepatic lipase activity caused by reduced levels of circulating estrogens (28). They might also be related with aging and age-related lifestyle changes that have a significant impact on lipoprotein metabolism. sdLDL-C, a subclass of LDL-C, is reported to easily penetrate into the arterial wall and, being highly susceptible to oxidation, may exacerbate and perpetuate atherosclerosis. sdLDL-C was associated with incident CHD in a prospective study among Atherosclerosis Risk in Communities (ARIC) study participants (10). The sdLDL-C/LDL-C ratio reflects the ability to generate sdLDL-C from LDL-C and may be elevated due to the high activity of hepatic lipase, which is associated with a high risk of CVD. Current studies suggest that the elevated levels of sdLDL-C or sdLDL-C/LDL-C ratio is considered to be the most atherogenic and much stronger predictive factors of cardiovascular events compared with only LDL-C (10, 26, 29–31), even in individuals considered to be at a low risk for CVD based on their LDL-C levels (10, 12). As emerging biomarkers associated with a variety of diseases, the sdLDL-C and sdLDL-C/LDL-C ratio have also been considered as important determinants of atherosclerosis in postmenopausal women.

sdLDL-C/HDL-C is the ratio of a risk factor (sdLDL-C) and a protective factor (HDL-C). Our study described that the sdLDL-C/HDL-C in postmenopause and perimenopause women, respectively, was significantly higher than in premenopause women but was the highest in perimenopause women, which might be due to the HDL-C levels being the highest in postmenopause women. In the recent years, sdLDL-C and the ratio of sdLDL-C to other blood lipid indexes have been used as surrogate risk markers in several studies, showing a higher clinical value of higher specificity and sensitivity with the cardiovascular system than a single index (10, 29). An Ning et al. suggested that the increase of sdLDL-C and sdLDL-C/HDL-C indicated an increased risk of acute coronary syndrome(ACS), and they were independent risk factors for ACS (32). However, there are very few studies on the correlation between sdLDL-C/LDL-C, sdLDL-C/HDL-C, and menopause. Our results also showed that, after adjusting for confounding variables such as age, etc., postmenopause was significantly related to the increase in sdLDL-C concentration, indicating that sdLDL-C is a promising postmenopause risk biomarker independent of age, etc., which we should pay close attention to in a subsequent follow-up study.

Dyslipidemia in postmenopause women is usually characterized by increased TC, LDL-C, and TG, and slightly decreased HDL-C. However, the evidence on the relationship between menopausal status and TG and HDLC has been inconsistent. Some report an increase, some report a decrease, while others note no change (24, 33–36). Our study found that the TC, LDL-C, and TG levels were much higher in perimenopause and postmenopause women than in premenopause women. The TG level was the highest in perimenopause women, and the HDL-C level was the lowest in perimenopause women while highest in postmenopause women. However, a community-based cohort study on serum lipid profile changes during the menopausal transition in Chinese women (33) indicated that TG increased to peak in late perimenopause, while HDL-C appeared to have no relationship with menopause status. Based on a 24,085-population-based cohort study of women aged 40–54 years, Graff-Iversen et al. reported that the HDL-C concentration appeared to increase slightly in perimenopause (35). The change in lipid profile is related to the decrease in estrogen level (33). Endogenous estrogens can directly regulate the synthesis, clearance, and modification of lipoproteins in the liver and are an important regulator of lipid synthesis and oxidation and inhibit vascular cell proliferation, inflammation, and vasoconstriction in premenopausal women (34). The results of our study (30–69-year-old women) showed that the prevalence of dyslipidemia in postmenopause and perimenopause women was 79.9% and 74.7%, respectively, significantly higher than that in premenopause women (54.5%) and higher than those in the 2012–2014 Chinese population study (age 40–59 years old; the prevalence of dyslipidemia in postmenopause and premenopause women was 69.7% and 24.3%, respectively) (37), also higher than those in patients in the clinic of the climacteric and menopause in Mexico in 2013 (the prevalence of dyslipidemia in postmenopause and perimenopause women was 70.8% and 62.3%, respectively) (38). The differences in results may be due to the different survey year, sample race, size, age range, definition of menopause, years after menopause, etc.

Estrogens play a beneficial role in regulating adiposity and glucose metabolism. Obesity and insulin resistance are less common in young and middle-aged women but are more prevalent in women over 60 years of age (39). The absence of estrogens after perimenopause has been suggested to be a cause of predominant abdominal fat accumulation and increased insulin resistance (40, 41). Donato GB et al. (42) stated that postmenopause women were five times more possible to have central obesity than premenopause women, even after controlling for BMI and other confounding factors. BMI increased in menopausal transition women but was not independently associated with central obesity. Our study also proved that WC, FBG, and HbA1c increased gradually and significantly in premenopause, perimenopause, and postmenopause women, while the median BMI remained almost the same among all groups with no significant difference. However, not all women deficient of estrogen become obese or develop insulin resistance. Estrogen deficiency in postmenopause women might increase blood pressure (6). Our results showed that DBP and SBP displayed gradually and significantly increasing trends among the premenopause, perimenopause, and postmenopause groups. However, Graff-Iversen et al. reported that menopausal status was not associated with adverse development of blood pressure and body weight (35). The heterogeneity of host genotype may play a role in them.

Perimenopause is a physiological transition period that every woman has to go through. During this period, many women suffer with different degrees of sleep disturbances and adverse moods due to the decline of estrogen levels caused by ovarian function decline as well as family and social factors (43). In our study, postmenopause women had the lowest proportion of good sleep quality (47.0%), followed by perimenopause women (55.2%), indicating that we should focus on sleep problems during health management in perimenopause and postmenopause women. Perimenopause women had the highest rate of alcohol consumption as well as dissatisfaction with life, which might be due to the fact that they have difficulty to accept in a short time the pathophysiological changes caused by the decline in estrogen levels. Berent-Spillson et al. (44) suggested that an interaction between metabolic and hormonal factors might influence emotion regulation and lead to a significantly increased risk for depression during menopause. Therefore, early screening of depression symptoms in perimenopause and postmenopause women and intervention for some severe cases are beneficial to improve the quality of life of middle-aged and elderly women.

There were significant differences in the number of pregnancies, age of menarche, cesarean, oral contraceptive, cumulative years of oral contraceptive use, IUD use, hysterectomy, and hemoglobin among the premenopause, perimenopause, and postmenopausal groups. For the postmenopause group (mainly 55+ years old), that is, women born before the 1970s, many of their family were poor then and they were malnourished, with a slower female development and their age of menarche the latest. In their childbearing years (before the 1990s), cesarean sections were rarely performed due to undeveloped medical science, so the proportion of cesarean cases was extremely low (4.5%). Furthermore, 94.6% of them never used oral contraceptives, and 42.4% never used an IUD. However, they had fewer pregnancies than the premenopause group, and the reason might be that they were more likely to use condoms. Although among premenopause women who were mainly born in the 1980s—their economic level had greatly improved since China’s reform and opening-up—64.7% used IUD and 20.6% took oral contraceptive, they had more times of pregnancies perhaps because they caught up with the universal two-child policy. The gradual increase in hemoglobin among the groups might be due to the decrease or cessation of menstruation caused by the decrease in estrogen levels, which, in turn, affected iron metabolism and led to an increase in iron reserves and promoted hemoglobin synthesis.

There were several limitations in our study. Firstly, it was a cross-sectional study, and we could describe associations while no causal relationship could be drawn. Secondly, there might be certain information bias when only one measurement of sdLDL-C was used since it was a dynamic change index. Thirdly, diet and genes were not considered as well as smoking (there were only seven smoking subjects—no statistically analysis could be performed), which might be confounding factors affecting the authenticity of the conclusion. Fourthly, birth control drugs/medications might affect blood lipids; however, only oral contraceptive was considered in our study. In addition, since there were only 87 perimenopause women (4.2% of the overall sample), caution is thus required in interpreting the results in subgroups of small sample size. More information should be collected and a larger sample size is recommended in our further study to compensate for these limitations. Lastly, the history of female fertility, such as the age of menarche and menopause, was self-reported by the subjects, who might have a certain recall bias.

Our study had several strengths. It was a cross-sectional survey with a large sample size and good representation based on community adults aged 30–69 years. Moreover, all of the examinations and questionnaires were measured and investigated by trained professionals in accordance with standard procedures, with good authenticity. Furthermore, excluding patients who had been diagnosed with type 2 diabetes mellitus (T2DM), who took lipid-lowering drugs and who used hormone replacement therapy, could exclude the influence of hypoglycemic, lipid-lowering drugs, and hormone on the study results.

## 5 Conclusions

After controlling for confounding factors, elevated sdLDL-C level was found to be significantly associated with postmenopause and independent of chronological aging. The results support that sdLDL-C is a promising risk biomarker for menopausal transition. In addition, the prevalence of dyslipidemia was much higher in postmenopause women than in premenopause women, so sdLDL-C combined with lipid monitoring is of great potential value for women’s healthcare and prevention of cardiovascular events. Further analysis, including long-term follow-up and the incorporation of dietary and genetics data, is warranted to robustly confirm our observed conclusions.

## Data Availability

The raw data supporting the conclusions of this article will be made available by the authors, without undue reservation.

## Glossary

sdLDL-C: small dense low-density lipoprotein cholesterol
T2DM: type 2 diabetes mellitus
LDL: low-density lipoprotein
FBG: fasting blood glucose
TC: total cholesterol
TG: triglyceride
LDL-C: low-density lipoprotein cholesterol
HDL-C: high-density lipoprotein cholesterol
HbA1c: glycated hemoglobin A1c
WC: waist circumference
SBP: systolic blood pressure
DBP: diastolic blood pressure
BMI: body mass index
CDC: Center for Disease Control and Prevention
OR: odds ratio
IR: insulin resistance
CVD: cardiovascular diseases
SWAN: Study of Women’s Health Across the Nation
Apo B: apolipoprotein B
ACS: acute coronary syndrome
CHD: coronary heart disease
IUD: intrauterine device
ARIC: atherosclerosis risk in communities
VLDL: very-low-density lipoprotein
CETP: cholesteryl ester transfer protein
